# Laser-Induced Electrochemical Biosensor Modified with Graphene-Based Ink for Label-Free Detection of Alpha-Fetoprotein and 17β-Estradiol

**DOI:** 10.3390/polym16142069

**Published:** 2024-07-19

**Authors:** Ridma Tabassum, Pritu Parna Sarkar, Ahmed Hasnain Jalal, Ali Ashraf, Nazmul Islam

**Affiliations:** 1 Graduate Research Assistant, The University of Texas Rio Grande Valley, Edinburg, TX 78539, USA; ridma.tabassum01@utrgv.edu (R.T.); prituparna.sarkar01@utrgv.edu (P.P.S.); 2 Department of Electrical & Computer Engineering, The University of Texas Rio Grande Valley, Edinburg, TX 78539, USA; ahmed.jalal@utrgv.edu; 3 Department of Mechanical Engineering, The University of Texas Rio Grande Valley, Edinburg, TX 78539, USA; ali.ashraf@utrgv.edu

**Keywords:** printable sensor design, flexible sensor applications, sensor coatings

## Abstract

In this research, a novel electrochemical biosensor is proposed based on inducing graphene formation on polyimide substrate via laser engraving. Graphene polyaniline (G-PANI) conductive ink was synthesized by planetary mixing and applied to the working zone of the developed sensor to effectively enhance the electrical signals. The laser-induced graphene (LIG) sensor was used to detect alpha-fetoprotein (AFP) and 17β-Estradiol (E2) in the phosphate buffer saline (PBS) buffer and human serum. The electrochemical performance of the biosensor in determining these biomarkers was investigated by differential pulse voltammetry (DPV) and chronoamperometry (CA). In a buffer environment, alpha-fetoprotein (AFP) and 17β-Estradiol detection range were 4–400 ng/mL and 20–400 pg/mL respectively. The experimental results showed a limit of detection (LOD) of 1.15 ng/mL and 0.96 pg/mL for AFP and estrogen, respectively, with an excellent linear range (R^2^ = 0.98 and 0.99). In addition, the designed sensor was able to detect these two types of biomarkers in human serum successfully. The proposed sensor exhibited excellent reproducibility, repeatability, and good stability (relative standard deviation, RSD = 0.96%, 1.12%, 2.92%, respectively). The electrochemical biosensor proposed herein is easy to prepare and can be successfully used for low-cost, rapid detection of AFP and E2. This approach provides a promising platform for clinical detection and is advantageous to healthcare applications.

## 1. Introduction

Estradiol (ESD), (1,3,5 (10)-estratrien), also known as 17-β-Estradioln (E2), is a naturally occurring steroid hormone synthesized from cholesterol in the human body. It is essential for the development and maintenance of female sexual characteristics [1]. This hormone, also utilized for birth control, has gained popularity in modern society and accounts for 16% of the selected contraceptive methods for preventing pregnancy [2]. For childbearing-aged women and postmenopausal women, the E2 level typically falls. Considering the daily excretions by humans, the associated dilution factor, and previous measurements, it was estimated that picograms per milliliter (pg/mL) levels are anticipated to be present in aqueous environmental samples [3]. However, since rivers and lakes are the ultimate receivers of steroid hormones, the E2 levels in water bodies are rapidly increasing. In the United States, a survey revealed concentrations ranging from 5 to 112 ng/L, whereas in China and France, concentrations range from 0.64 to 55.3 ng/L and 1.1 to 3.0 ng/L, respectively [2]. The elevated level of E2 may impact the reproductive health of both humans and animals through the food chain. This can lead to abnormalities in the growth and function of the male reproductive system, contribute to a decline in male birth rates, and may induce an increase in the incidence of tumors [4].

Cancer can occur due to several factors like genetics, work, rest, environment, etc. [5]. In 2020, almost 19.3 million new cancer cases were reported, and almost 10.0 million cancer deaths occurred worldwide [6]. Among all the cancers, liver cancer tends to recur frequently. Among men, it is the fifth most common cause of death from cancer. According to cancer.net, an estimated 29,380 deaths occurred in the United States in 2023 due to liver cancer. This clearly means that the prognosis of liver cancer is significant. Hepatocellular carcinoma (HCC) constitutes 70–80% of all liver cancer cases. The most widely recognized and crucial biomarker for HCC is AFP [7]. It is a multifunctional glycoprotein of the albuminoid gene family with a dual regulatory role in both cancer and fetal activities. Normally, this protein is produced by the liver, yolk sac, and gastrointestinal tract in humans [8]. In healthy humans, the AFP level in serum is generally expected to be lower than 25 ng/mL. However, an elevation of AFP levels to 500 ng/mL could be indicative of HCC in patients [9]. Hence, the quantitative analysis of AFP concentration is crucial for early clinical diagnosis of liver cancer and long-term treatment.

In recent years, various techniques for the detection of AFP have been tested by various scientists and researchers. Foluke et al. [10] investigated the collaborative utilization of carbon black nanoparticles (CBNPs) and palladium nanoparticles (PdNPs) to create a nanohybrid construct for the fabrication of an electrochemical immunosensor aimed at detecting alpha-fetoprotein (AFP). Haotian Wu et al. [11] developed a complicated novel electrochemical biosensor integrating iron tetroxide (Fe_3_O_4_)/carboxylated carbon nanotubes (MWCNTs-COOH)/gold nanoparticles (AuNPs) for AFP detection, utilizing a solvothermal approach for nanocomposite synthesis and electrode modification with AFP antibodies via NHS/EDC immobilization. Various optical biosensors have been developed for the detection of AFP [12,13]. For optical biosensors, various methods like fluorescence [14], electrochemiluminescence [15,16], surface plasmon resonance (SPR), localized surface plasmon resonance (LSPR) [17], colorimetric [18], luminescence energy transfer (LET) [19], surface-enhanced Raman spectroscopy (SERS) [20], photoelectrochemical (PEC) [21], etc. are mostly used. Although these techniques exhibit excellent sensitivity and stability, they suffer from drawbacks such as high cost, time-consuming processes, elevated technical barriers, lack of portability, and bulkiness. Preeyanuch Supchocksoonthorn et al. [22] fabricated a polyaniline/carbon dot-coated glassy carbon electrode electrochemical sensor for the detection of 17β-estradiol. Auwal M. Musa et al. [23] developed a screen-printed electrode (SPE) modified with gold nanoparticle-decorated reduced graphene oxide–carbon nanotubes (rGO-AuNPs/CNT/SPE) which have a linear sensitivity of 0.05–1.00 µM. Apart from electrochemical methods, liquid chromatographic-mass spectrometric method (HPLC/MS) [24], liquid chromatography [25], capillary electrophoresis [26], UV spectrometry [27], etc. are used for the detection of estrogen. In our research, we provided a wide range of characteristics comparing with the other mechanisms that are provided in the literature. Thus, electrochemical biosensors are gaining popularity day by day since the surface of electrodes can undergo modification using a wide range of materials and nanoparticles, which enhance stability, improve conductivity, and increase surface area for optimal immobilization of biological recognition agents in biosensors [12,28,29]. The sensors are highly sensitive, portable, easy to fabricate, and cost-effective, making them ideal for point-of-care (POC) testing applications.

Since its discovery in 2004 [30], graphene has been the subject of intense study, revealing remarkable electronic properties and potential applications in biosensing. Its high surface area-to-volume ratio makes graphene-based sensors highly sensitive to environmental changes, enabling precise detection of analyte concentration. Additionally, graphene’s ability to be functionalized with specific receptors enhances both sensitivity and selectivity, enabling the detection of specific analytes such as DNA sequences or single nucleotide mutations. Novel sensing platforms have emerged, utilizing pristine and modified graphene combined with nanoparticles and polymers, enabling the immobilization of biomolecules like antibodies, DNA, and enzymes, resulting in highly sensitive and selective biosensors with diverse applications [31]. Graphene can be produced through various synthesis methods, including solid phase exfoliation, liquid phase exfoliation, electrochemical exfoliation, chemical reduction, thermal reduction, chemical synthesis, chemical vapor deposition, thermal pyrolysis, and epitaxial growth (using SiC) [32]. However, certain techniques are time-consuming, expensive, and require specialized equipment and personnel [33]. To synthesize graphene easily, Lin et al. [34] introduced a single-step, scalable method for fabricating and patterning porous graphene films featuring three-dimensional networks. This technique involves utilizing a CO_2_ infrared laser to treat commercial polymer films. Through pulsed laser irradiation, sp^3^-carbon atoms within the polymer are converted to sp^2^-carbon atoms, resulting in the formation of laser-induced graphene (LIG) with high electrical conductivity. LIG technology enables the creation of self-supported electrodes. The direct laser-scribing method has emerged as a promising technique for synthesizing three-dimensional (3D) graphene due to its high throughput, customizable patterning, cost-efficiency, and environmental friendliness. This noncontact laser process is simple, rapid, and precise, producing high-quality carbon-based materials with complex micro/nanostructures and high reproducibility [35]. The use of transient CO_2_ lasers on materials such as paper, wood, lignin, and thermoplastic polymers has shown promise, facilitating the synthesis of porous 3D graphene for applications including supercapacitors, catalysis, and electrochemical sensors [36,37].

In our paper, a simple, flexible, disposable, time, and cost-efficient G-PANI-coated laser-induced graphene (LIG) sensor is fabricated for the detection of estrogen and AFP. The performance of the sensor was analyzed via differential pulse voltammetry (DPV) and chronoamperometry (CA). Initially, we focused on just the detectability of the sensor for estrogen and AFP. Later, we tested the sensor for repeatability and reproducibility. The surface characterization of the sensor was evaluated using a scanning electron microscope (SEM), X-ray powder diffraction (XRD), and Fourier-transform infrared spectroscopy (FTIR). The limit of detection (LOD) of the sensor for estrogen is 0.96 pg/mL in buffer solution. The immunosensor is able to detect AFP with an LOD of 1.15 ng/mL. Furthermore, it exemplifies good electrochemical performance toward the detection of liver cancer proteins and estradiol.

## 2. Materials and Methods

### 2.1. Materials and Instruments

Kapton HN polyimide film (0.152 mm thickness), graphene nanoflakes (thickness: 2–10 nm thickness, diameter size: ~5 μm, specific surface area: 20 to 40 m^2^/g), polyaniline (CAS Number: 62-53-3), phytic acid (CAS No: 83-86-3), potassium hexacyanoferrate (II) trihydrate (CAS No: 14459-95-1), and potassium hexacyanoferrate (III) (CAS No: 13746-66-2), N-Hydroxysuccinimide (NHS) (CAS No: 6066-82-6), and N-(3-Dimethylaminopropyl)-N’-Ethylcarbodiimide Hydrochloride (EDC) (CAS No: 25952-53-8) were obtained from Sigma Aldrich (St. Louis, MO, USA). The polydimethylsiloxane (PDMS) layer was prepared using SYLGARD 184 Silicone Elastomer Base and Curing Agent in a ratio of 10:1. Recombinant Mouse alpha 1 Fetoprotein and Anti-alpha 1 Fetoprotein antibodies were purchased from Abcam (Boston, MA, USA) and 99% Potassium hexacyanoferrate (II) trihydrate Estradiol Elisa kit was purchased from Cayman (Ann Arbor, MI, USA) (Animal/Rabbit, Animal/Eel, Animal/Bovine origin). The silver ink and human serum (H4522) were acquired from Sigma-Aldrich (St. Louis, MO, USA). All the reagents were used as received without any further purification.

Glowforge Pro (CO_2_ Laser, 45 watts) (Glowforge, Seattle, WA, USA) was used to fabricate the LIG sensor. All electrochemical experiments were executed on Autolab Potentiostat (PGSTAT302N) (Metrohm, Riverview, FL, USA). The scanning electron microscopy (SEM) images were captured with a Zeiss field emission scanning electron microscope (Zeiss, Oberkochen, Germany). The FTIR data was obtained by Thermo Scientific Nicolet iS5 FTIR Spectrometer (Thermo Scientific, Waltham, MA, USA). A Hauschild SpeedMixer (SMART DAC 250-2000) (Hauschild SpeedMixer Inc., Farmington Hills, MI, USA) was used to acquire a uniform ink solution. For the ozonolysis treatment, the Novascan digital UV ozone system (OES-1000D-Ozone Elimination System) was used.

### 2.2. Synthesis of Graphene-Polyaniline Ink

Graphene–polyaniline ink (G-PANI ink) was obtained by combining 1 g of graphene nanoflakes (GNF) with 2 mL of polyaniline, 4 mL of phytic acid, and 6 mL of de-ionized (DI) water [1]. In order to achieve a uniform ink solution, we blended these chemical components utilizing the Hauschild SpeedMixer SMART DAC at a speed of 2500 RPM. Phytic acid was selected as the medium for ink preparation because it creates an acidic environment that increases electrical conductivity in polyaniline (PANI) [38].

### 2.3. Electrodes Fabrication

**Figure 1:**
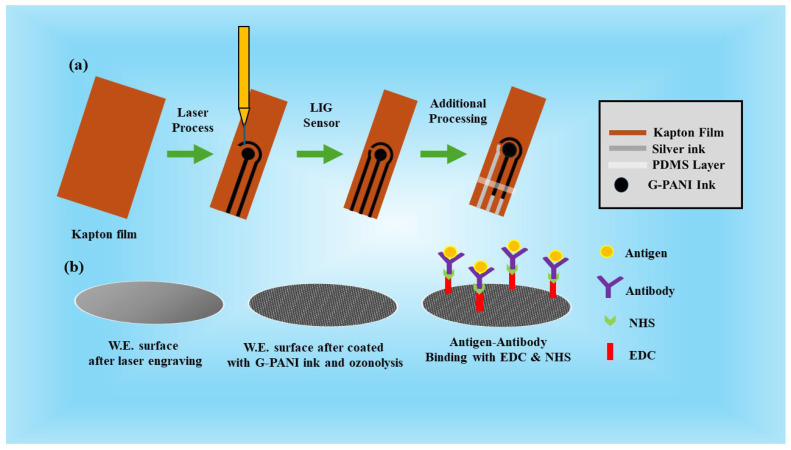
Schematic representation of (**a**) LIG sensor fabrication using laser engraving process on Kapton sheet and (**b**) immobilization steps on the working electrode surface.

The biosensor (size: L = 13.7 mm, W = 7.2 mm, working electrode diameter = 3.7 mm) was designed using AutoCAD software (2023). The pattern of the graphene-based sensor was fabricated on Kapton film (thickness of 152 µm) using a direct laser writing process using Glowforge Pro machine (CO_2_ laser, 10.6 µm wavelength). The engraving process was performed at a speed of 650 and a precision power of 50% of the full power (45 watts). The LIG sensors were fabricated in a three-electrode configuration under ambient conditions, as shown in Figure 1. In this process, sp^3^ carbon in polyimide is converted into a highly conductive hybridized sp^2^ graphene structure. After the fabrication process, the silver ink was manually applied to create reference electrodes and contact pads. The objective of this painting is to ensure better electrical contact and precise measurements. Afterward, the working electrode (W.E.) was modified with 5 µL of graphene-conductive polyaniline ink. Then, a thin hydrophobic PDMS layer was employed between the working area and contact pads to prevent the flow of analyte solution from the detection zone to contact pads. Afterward, the fabricated sensors were kept at room temperature (~25°) overnight to dry.

### 2.4. Immobilization of Antibody onto the Working Electrode Surface

A specific procedure was followed for surface immobilization. Initially, the laser-engraved electrode underwent an ozonolysis treatment (3 min of exposure at 40 °C) to introduce the -COOH-functional group onto the sensor surface. Subsequently, approximately 10 µL of 0.4M EDC solution was applied to the working electrode (W.E.) region. The sensor was left in a dark environment for 2 h. EDC is a chemical reagent commonly used for activating carboxylic acid groups (-COOH) on the surface. Following this, 10 µL of 0.4M NHS solution was introduced to the working electrode, and the electrode was again kept in darkness for 2 h. Afterward, a fixed volume of 10 μL of antibody (10 μL AFP antibody for the development of an AFP biosensor or 10 μL of E2 antibody for the development of an E2 biosensor) was left for 1 h over the electrode surface for conjugating with NHS molecules. After this step, the sensor was preserved at 4 °C until it was used.

### 2.5. Electrochemical Measurement

The DPV and CA measurements were performed using Autolab Potentiostat-PGSTAT302N, respectively. The potentiostat can handle a maximum current range of 2A, and Nova 2.1 software was integrated with the potentiostat to conduct electrochemical measurements. Analytical curves were generated using data collected after applying 10 μL of various concentrations of AFP/E2 antigen over the biosensors, allowing these biomarkers to react during an optimized time of 20 min.

## 3. Result and Discussion

### 3.1. Characterization of Prepared LIG/G-PANI

**Figure 2:**
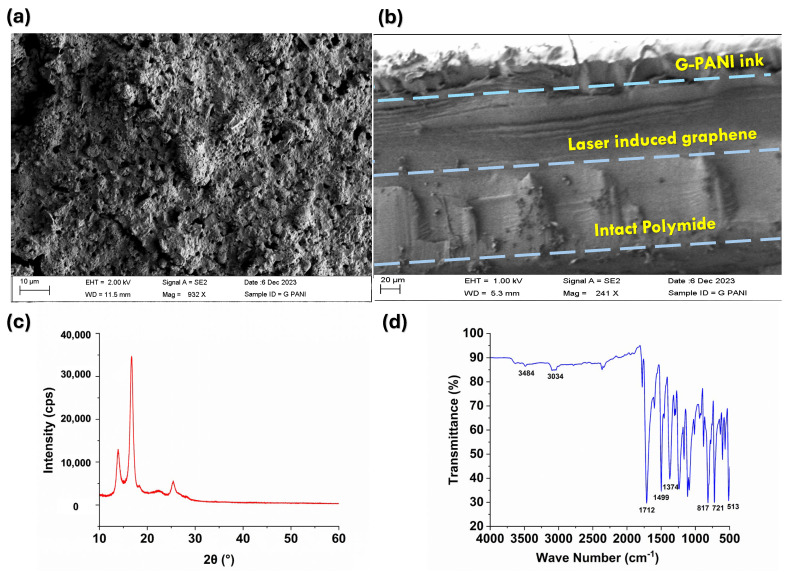
(**a**) SEM image of LIG/G-PANI sensor surface; (**b**) Cross-sectional view of the sensor coated with G-PANI ink; (**c**) XRD of the LIG induced on the PI substrate; (**d**) FTIR spectra of LIG/G-PANI.

Scanning electron microscopy (SEM) was used to characterize the morphology of the surface of a laser-induced graphene sensor modified with G-PANI conductive ink. Figure 2a shows a uniform distribution of G-PANI nanocomposites clearly observed on the surface of the LIG sensor. The surface morphology of the modified electrode exhibits a porous texture resulting from the rapid liberation of gaseous products during laser treatment [34]. The porous structure displayed a complex three-dimensional network with a significant surface area, potentially augmenting detection sensitivity [39]. The cross-sectional image is shown in Figure 2b. In this image, three distinct regions are discernible: the unaffected polyimide base, the laser-induced graphene area, and the G-PANI ink layer on top. After laser treatment, roughly 40% of the Kapton film was converted into laser-induced graphene.

Moreover, the crystallographic analysis of the fabricated LIG/G-PANI sensor was conducted using an X-ray diffractometer (XRD) with a scanning range for 2θ values 10 to 60° (Figure 2c). The XRD pattern measurements reveal the structural characteristics of the prepared samples. The crystalline peaks appeared at 16.7°, 22.4°, and 25.4°, representing the (0 1 1), (0 2 0), and (2 0 0) crystal planes of PANI [40,41,42]. The peak centered at 2θ = 25.9° suggests a high level of graphitization. According to the XRD data, the laser-induced graphene was successfully prepared.

The chemical bonds present in graphene polyaniline (G-PANI) conductive ink were investigated by FTIR spectra, as illustrated in Figure 2d. The peak observed at 3484 cm^−1^ is related to the N-H stretching band [41]. The band at around 3034 cm^−1^ is associated with C-H stretching vibration [42]. In G-PANI, peaks characteristic of the benzene and quinone structures were present. Generally, the peaks at 1712 cm^−1^ and 1499 cm^−1^ correspond to the stretching vibration of C=C on the quinone ring and the benzene ring, respectively [43]. The band stationed at 1374 cm^−1^ is assigned to C-N stretching vibrations of secondary aromatic amine [44]. The peaks that appeared at 817–513 cm^−1^ are attributed to the bending of the aromatic C-H bond [43,45].

### 3.2. Alpha-Fetoprotein Detection

**Figure 3:**
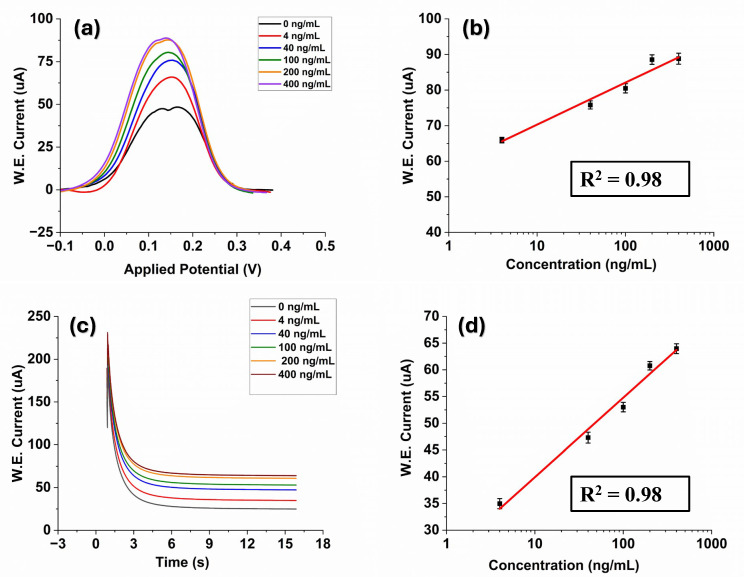
(**a**) DPV current responses of the proposed immunosensor at different AFP concentrations (4–400 ng/mL); (**b**) The calibration curves between DPV peak current response and different AFP concentrations; (**c**) The chronoamperometric response with various concentrations of AFP; (**d**) The linear relationship between the current value of the chronoamperometric response at 15.5 s and the AFP concentrations.

In this study, we evaluated the performance of the prepared laser-engraved Kapton sensor modified by graphene polyaniline ink by detecting liver cancer proteins. Our evaluation involved employing the DPV technique due to its high sensitivity in electrochemical experiments. After the immobilization process, the surface of the biosensor was exposed to various concentrations of AFP. The concentration of the stock solution of antigen is 0.4 mg/mL. The stock solution was diluted with phosphate-buffered saline (PBS) (pH = 7.4) and prepared different desired concentrations from the range of 4 ng/mL to 400 ng/mL using the serial dilution method. A 20-min incubation duration was implemented to provide ample time for the antigen solution to reach and form bonds with the immobilized antibody. It can be observed from Figure 3a that the DPV signals are capable of detecting AFP. The obtained DPV oxidation peak current rises as the sample concentration range increases, spanning from 4 ng/mL to 400 ng/mL. We selected this range as an AFP level between 0 ng/mL and 10 ng/mL is normal for adults. An extremely high level of AFP in the blood—greater than 400 ng/mL—could be a sign of liver tumors [46]. Moreover, it can be observed that the signal value exhibits a gradual slowdown with increasing concentration. This may occur due to the electrode reaching saturation at higher concentrations [5].

The DPV analysis conducted across varying concentrations of AFP demonstrated a linear relationship between the peak current and the logarithmically plotted concentrations with a correlation coefficient of R^2^ = 0.98, and the linear regression equation was $I\;\left(uA\right)=58.48+11.83\;log\;$(Figure 3b). The limit of detection was also calculated to be ~1.15 ng/mL using the formula LOD = σ/S where (σ is the standard deviation of the response and S is the slope = 0.13 × slope of semi-log plot)) [47]. The obtained result is compared to previously reported AFP electrochemical biosensors in the literature (Table 1). The proposed LIG sensor exhibits relatively high analytical performance and an LOD for the determination of AFP.

Chronoamperometry involves studying the current response changes over time under potentiostatic control [54]. CA studies of AFP were conducted by fixing the working electrode potential at 0.28 V, and Figure 3c shows the CA analyses of AFP standard solutions at different concentrations (4, 40, 100, 200, 400 ng/mL in PBS at pH 7.4). It could be observed that the current had approached a plateau in a short period of 15 s. Increasing concentrations of AFP resulted in an increase in the current response due to the binding interaction between the antibody–antigen of AFP on the electrode surface, which can be seen in Figure 3c. The electrical signal response difference had a good linear relationship with the AFP antigen concentration with a coefficient of determination R^2^ = 0.98 where the regression equation was I (uA)=25.06+14.86 logC (Figure 3d). The LOD was 1.18 ng/mL. These results sufficiently indicate that our developed sensor can successfully detect AFP in PBS.

### 3.3. Estrogen Detection

Similar to AFP detection, the designed biosensor was utilized to determine standard E2 solution with varying concentrations to assess its performance. The stock antigen solution (400 ng/mL) was serially diluted with PBS (pH = 7.4) to prepare different concentrated solutions. The prepared working electrodes were incubated in different concentrations of E2 antigen solutions under optimized experimental conditions. Both DPV and CA methods were used for the electrochemical characterization.

**Figure 4:**
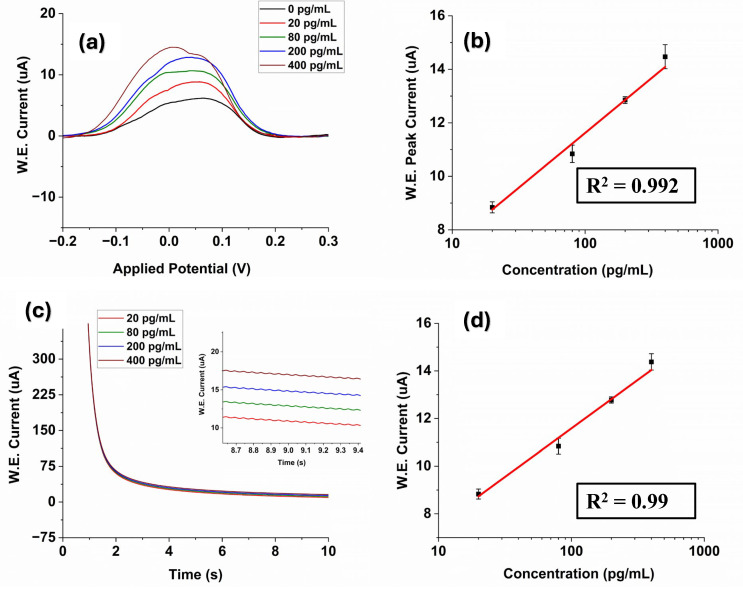
(**a**) Differential pulse voltammograms of 17 β-estradiol over the concentration range of 20–400 pg/mL in PBS solution; (**b**) The calibration curve of the DPV outputs and 17 β-estradiol concentrations; (**c**) Chronoamperometric measurements of estradiol 17 at various concentrations (20–400 pg/mL), the enlarged view is in the inset; (**d**) The calibration curve of the CA outputs and 17 β-estradiol concentrations.

The measurement results for a series of different concentrations of E2 ranging from 20 pg/mL to 400 pg/mL are shown in Figure 4a. The DPV current response of the fabricated G-PANI/LIG biosensor increased as the E2 concentration rose (Figure 4a). It is consistent with the fact that the more E2 exists in the system, the more formation of a stable immunocomplex between antigen and antibody will occur, resulting in an increase in the current. In addition, an evident linear relationship identified between DPV current response and the logarithm of AFP concentration is observed from the calibration curve. The DPV regression equation was I(μA)=3.42+4.09 logC (Figure 4b) with a correlation coefficient (R^2^) of 0.992. The LOD was estimated to be 0.96 pg/mL.

Table 2 summarizes the performance comparison between the previous reports for estrogen detection in literature and our immunosensor. Researchers adopted various structures and methods for estrogen detection. Graphene–polyaniline (GR–PANI) composites and carboxylated graphene oxide (GO)-based immunosensors showed a detection range of 0.04–7.00 ng/mL and a LOD of 0.02 ng/mL [55]. Yang et al. developed a paper-based immunosensor modified with multi-walled carbon nanotubes nanocomposites for detection of 17β-E2 (range: 0.01–100 ng/mL and LOD: 10 pg/mL) [56] CoFe_2_O_4_/graphene nanohybrid (CoFe_2_O_4_/rGO) was also used to detect estrogen in a range of 0.01–18.0 ng/mL (LOD = 3.3 pg/mL) [57]. A nanostructured magnetic molecularly imprinted polymer (range: 13.6–2720 ng/mL, LOD: 5440 pg/mL) [58] label-free electrochemical aptasensor (range: 0.019–2.7 ng/L, LOD: 0.014 ng/mL) [59] was also employed for estradiol detection.

Furthermore, chronoamperometric analysis was performed to test the analytical performance of the proposed G-PANI/LIG biosensors. Chronoamperometric records for E2 detection on the LIG sensor surface were performed at the potential of 280 mV at variable estrogen concentrations in PBS (pH = 7.4). The chronoamperograms for the determination of E2 in the concentration range of 20–400 pg/mL are represented in Figure 4c. It can be observed that the current of the G-PANI modified electrode decreases rapidly at the initial stage within 8 s and then quickly tends to a stable value. Further, the current of laser-induced graphene obviously increases with increasing concentrations of E2, suggesting that there is an increase in the rate of the electrochemical reaction on the electrode surface. The amperometric response for the estradiol detection showed a linear calibration curve, which was obtained by plotting the current increase versus the logarithm concentration of the estrogen, as displayed in Figure 4d. The linear interval can be expressed by the equation $I\left(uA\right)=3.43+4.07\;logC$ (R^2^ = 0.992) (Figure 4d), where current is in microampere (uA). The LOD was estimated to be 0.98 pg/mL.

### 3.4. Detection of Alpha-Fetoprotein and 17β-Estradiol in Human Serum

**Figure 5:**
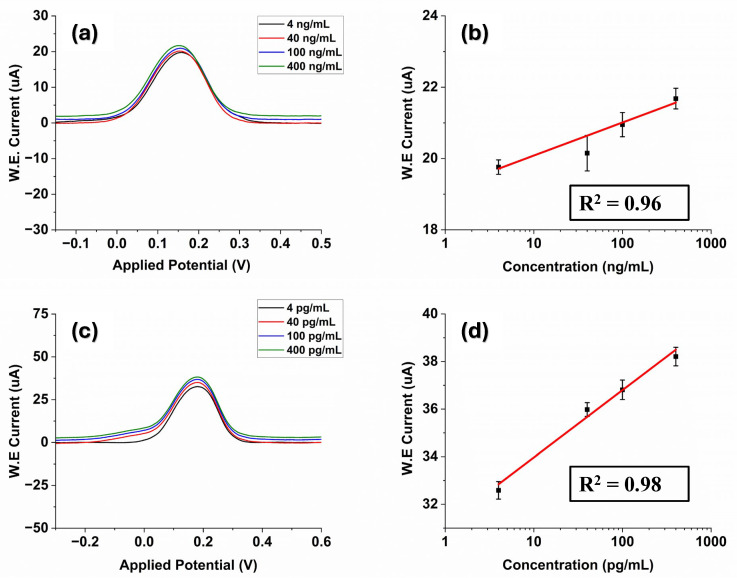
(**a**) DPV measurements of AFP detection in human serum from 4 to 400 ng/mL; (**b**) Linear relationship between DPV peak current and different AFP concentrations; (**c**) DPV measurements of estradiol 17 at various concentrations in human serum; (**d**) Linear regression curve of DPV responses vs. estrogen concentrations.

The presence of complex substances in human serum could hinder the detection of AFP and E2 in serum. The performance of the fabricated biosensor in more complex biological environments was studied by detecting AFP in human serum (H4522). AFP antigen at varying concentrations (4–400 ng/mL) in human serum samples was assayed with DPV measurements, and the results are shown in Figure 5a. Similar to the test in PBS buffer solution, the DPV current response of the proposed immunosensor increased as AFP concentration was on the rise. However, the change in the peak current value was not very significant. This may happen due to the complex nature of the serum; this complexity may impact the electron transfer process and the availability of AFP for interaction at the electrode surface. Figure 5b shows the corresponding calibration curve of DPV measurements in serum based on results shown in Figure 5a. As can be seen from Figure 5b, the current response was linearly proportional to the AFP concentration. The regression equation was I(uA)=0.927+19.15 logC (Figure 5b) with a correlation coefficient of 0.9628. The LOD was 1.76 ng/mL. Six repeated measurements of AFP at a concentration of 4.0 ng/mL were performed to evaluate precision. The resulting relative standard deviation (R.S.D.) was found to be 1.04%.

Furthermore, the biosensor was applied in the detection of E2 in human serum. The E2 concentration, as measured by the biosensor, ranged from 4 to 400 pg/mL. Figure 5c indicates that the DPV peak increased with the rising concentration of E2. As shown in Figure 5d, the peak current increment values were linearly dependent on the E2 concentrations with correlation equations of I(uA)=31.14+2.83 logC (R^2^ = 0.98) (Figure 5d), and the LOD of 1.32 pg/mL was obtained. It can be clearly seen from the obtained results that our method showed good analytical performance even in complex biological environments.

### 3.5. Evaluation of Reproducibility, Repeatability, and Stability of the Biosensor

**Figure 6:**
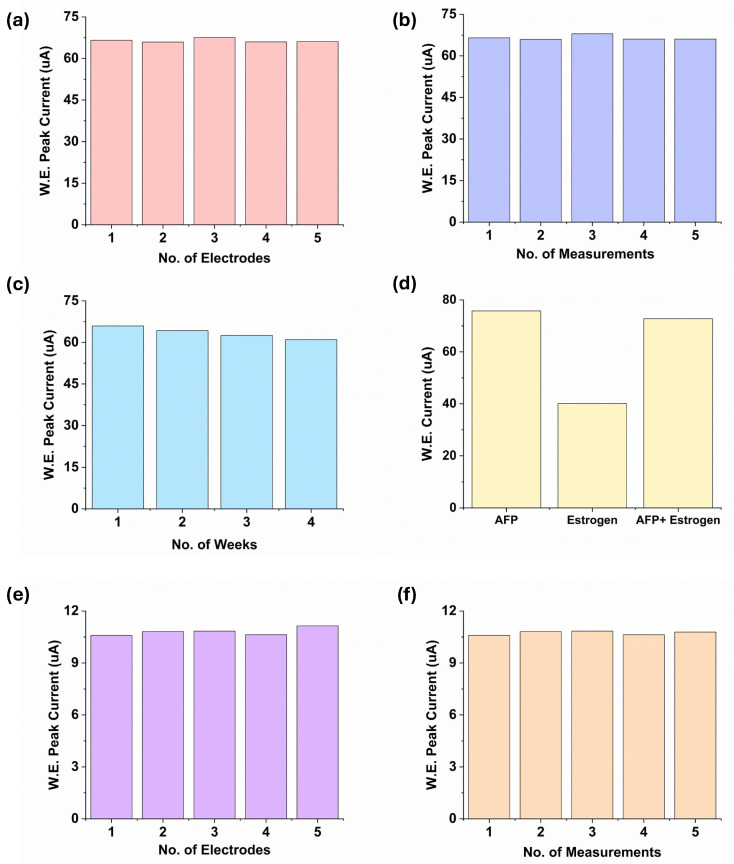
The histograms of the peak current of the immunosensor. (**a**) Reproducibility; (**b**) Repeatability; (**c**) Stability; (**d**) Selectivity of the developed LIG sensor for AFP detection; (**e**) Reproducibility; (**f**) Repeatability test for E2 detection.

Reproducibility, repeatability, and stability are also important metrics for evaluating the biosensor. In this experiment, five sets of biosensors were prepared under identical experimental conditions to assess the reproducibility of the designed biosensor. A total of 4 ng/mL AFP was used as the concentration for the reproducibility test of the proposed biosensor. The RSD was calculated as 0.95%, indicating that the biosensor prepared by the proposed strategy has excellent reproducibility (Figure 6a). The negligible variation is suspected to result from the biosensor manufacturing process. In addition, the repeatability of the LIG sensor was also measured (Figure 6b). Repeatability was evaluated based on five measurements conducted in the presence of 4 ng/mL AFP antigen solution on the sensor surface. Based on the obtained results, the proposed sensors exhibit good repeatability with an RSD of 1.12%.

The stability of storage plays a pivotal role in making the biosensor more applicable for bio-analysis. The stability of the fabricated sensor was also examined over the four weeks (Figure 6c). At the various storage periods between four weeks, the designed sensor was employed to detect the same concentration (4 ng/mL) of AFP and was stored in a 4 °C refrigerator when it was not in use. The analytical performance showed no noticeable decline during the initial storage period. In the third week, the current response reached about 94.74% of the original value. After four weeks, the immunosensor retained its bioactivity and showed satisfactory storage stability with a good RSD value of 2.92%. In this study, 4 ng/mL AFP was mixed with 4 pg/mL 17β-estradiol to assess the selectivity of the developed biosensor. As shown in Figure 6d, when the biosensor was tested with only a 4 pg/mL estrogen solution, the current was significantly reduced because the sensor was immobilized with the AFP antibody. However, no significant changes in current were observed when interference substances were added, demonstrating the high selectivity of the biosensor.

Furthermore, the reproducibility and repeatability of the proposed biosensor were examined for 17β-estradiol detection. The obtained results are shown in Figure 6e,f. The relative standard deviations of the response current for immunosensors were around 0.93% (for repeatability) and 1.80% (for reproducibility), revealing good accuracy and excellent reproducibility of the immunosensor. Overall, this electrochemical biosensor possesses high repeatability, excellent reproducibility, and favorable stability for the accurate detection of AFP and estrogen.

## 4. Conclusions

The goal of this research work is to develop an efficient, cost-effective, and easy-manufactured electrochemical biosensor utilizing laser-induced graphene for the detection of liver cancer diagnosis (AFP) and E2. The proposed method makes the process of detecting AFP and E2 simpler, faster, and cheaper. Furthermore, the working zone of the fabricated sensor was modified with graphene-based conductive inks to increase the analytical performance of the immunosensor. The surface morphology was examined using SEM characterization, and the XRD pattern represents the structural characteristics of the LIG/G-PANI sensor. The developed sensor demonstrates excellent performance in liver cancer biomarkers and E2. Under optimal conditions, the immunosensor exhibits a good detection limit within the linear sensing range of 4–400 ng/mL for AFP and 20–400 pg/mL for E2, with R^2^ values of 0.98 and 0.99, respectively. Moreover, these LIG sensors have been successfully applied to the detection of liver cancer proteins and estradiol in human serum. The LOD of AFP in PBS is 1.15ng/mL, and for E2, it is 0.96 pg/mL. In serum, the LOD for AFP is 1.76 ng/mL, and for E2, it is 1.32 pg/mL. The proposed sensor demonstrates satisfactory repeatability, reproducibility, and stability performance. Future work will also involve the detection of these biomolecules from actual samples from affected patients.

## Figures and Tables

**Table 1:** Comparison of the analytical performance of different methods toward AFP.

**Electrode**	**Technique**	**Linear Range (ng/mL)**	**LOD**	**Reference**
MIP/PDA/GS-Au/PTh/GCE	DPV	0.001–1000	3.7 pg/mL	[48]
Fe_3_O_4_/MWCNTs-COOH/AuNPs	DPV	0.001–10,000	1.09 pg/mL	[11]
Fe_3_O_4_@Au@chitosan	Amperometry	10–8000	1.78 ng/mL	[10]
Au/AET/PAMAM	CV	5–500	3 ng/mL	[49]
Au/PA	CV	5–80	3.7 ng/mL	[50]
Self–assembled monolayers AuNPs/HRP	-	15–350	5 ng/mL	[51]
AuNPs/PGNR	DPV	5–60	1 ng/mL	[52]
Pd nanoplates	SWV	0.01–75.0	4 pg/mL	[53]
G-PANI/LIG	DPV	4–400	1.15 ng/mL	This work

**Table 2:** Comparison of 17β-estradiol detection performance for different biosensors.

Electrode	Technique	Linear Range	LOD	Reference
Ag/PAMAM–Au/GR–PANI/GCE	DPV	0.04–7.00 ng/mL	0.02 ng/mL	[55]
NH_2_-SWCNT/NMB/AuNP	DPV	0.01–500 ng/mL	5 pg/mL	[60]
MWCNTs/THI/AuNPs nanocomposites	DPV	0.01–100 ng/mL	10 pg/mL	[56]
CoFe_2_O_4_/rGO	Amperometry	0.01–18.0 ng/mL	3.3 pg/mL	[57]
Fe_3_O_4_-MIP	DPV	13.6–2720 ng/mL	5440 pg/mL	[58]
MCH/Au	DPV	0.019–2.7 ng/L	0.014 ng/L	[59]
MWCNTs/AuNP	SWV	0.001–1 ng/mL	1 pg/mL	[61]
G-PANI/LIG	DPV	0.02–0.4 ng/mL	0.96 pg/mL	This work

## Data Availability

The original contributions presented in the study are included in the article, further inquiries can be directed to the corresponding author.

## References

[1] Butler D., Guilbault G.G. (2006). Disposable amperometric immunosensor for the detection of 17-β estradiol using screen-printed electrodes. Sens. Actuators B Chem..

[2] Ghosh D., Rahman M.S., Ashraf A., Islam N. (2023). Graphene Nanoparticle Modified Laser Engraved Kapton Sensor for Environmental Estrogen Detection. Volume 12: Micro- and Nano-Systems Engineering and Packaging.

[3] Johnson A.C., Belfroid A., Di Corcia A. (2000). Estimating steroid oestrogen inputs into activated sludge treatment works and observations on their removal from the effluent. Sci. Total Environ..

[4] Yildirim N., Long F., Gao C., He M., Shi H.-C., Gu A.Z. (2012). Aptamer-Based Optical Biosensor For Rapid and Sensitive Detection of 17β-Estradiol In Water Samples. Environ. Sci. Technol..

[5] Yen Y.-K., Huang G.-W., Shanmugam R. (2024). Laser-scribing graphene-based electrochemical biosensing devices for simultaneous detection of multiple cancer biomarkers. Talanta.

[6] Sung H., Ferlay J., Siegel R.L., Laversanne M., Soerjomataram I., Jemal A., Bray F. (2021). Global Cancer Statistics 2020: GLOBOCAN Estimates of Incidence and Mortality Worldwide for 36 Cancers in 185 Countries. CA Cancer J. Clin..

[7] Heiat M., Negahdary M. (2019). Sensitive diagnosis of alpha-fetoprotein by a label free nanoaptasensor designed by modified Au electrode with spindle-shaped gold nanostructure. Microchem. J..

[8] Upan J., Youngvises N., Tuantranont A., Karuwan C., Banet P., Aubert P.-H., Jakmunee J. (2021). A simple label-free electrochemical sensor for sensitive detection of alpha-fetoprotein based on specific aptamer immobilized platinum nanoparticles/carboxylated-graphene oxide. Sci. Rep..

[9] Li G., Li S., Wang Z., Xue Y., Dong C., Zeng J., Huang Y., Liang J., Zhou Z. (2018). Label-free electrochemical aptasensor for detection of alpha-fetoprotein based on AFP-aptamer and thionin/reduced graphene oxide/gold nanoparticles. Anal. Biochem..

[10] Olorundare F.O.G., Sipuka D.S., Sebokolodi T.I., Kodama T., Arotiba O.A., Nkosi D. (2023). An electrochemical immunosensor for an alpha-fetoprotein cancer biomarker on a carbon black/palladium hybrid nanoparticles platform. Anal. Methods.

[11] Wu H., Zhang G., Yang X. (2023). Electrochemical immunosensor based on Fe_3_O_4_/MWCNTs-COOH/AuNPs nanocomposites for trace liver cancer marker alpha-fetoprotein detection. Talanta.

[12] Mohammadinejad A., Kazemi Oskuee R., Eivazzadeh-Keihan R., Rezayi M., Baradaran B., Maleki A., Hashemzaei M., Mokhtarzadeh A., De La Guardia M. (2020). Development of biosensors for detection of alpha-fetoprotein: As a major biomarker for hepatocellular carcinoma. TrAC Trends Anal. Chem..

[13] Kal-Koshvandi A.T. (2020). Recent advances in optical biosensors for the detection of cancer biomarker α-fetoprotein (AFP). TrAC Trends Anal. Chem..

[14] Lu C., Wei D., Li G. (2019). A fluorescence turn-on biosensor based on gold nanoclusters and aptamer for alpha fetoprotein detection. IOP Conf. Ser. Earth Environ. Sci..

[15] Wang X., Gao H., Qi H., Gao Q., Zhang C. (2018). Proximity Hybridization-Regulated Immunoassay for Cell Surface Protein and Protein-Overexpressing Cancer Cells via Electrochemiluminescence. Anal. Chem..

[16] Cui M., Yu R., Wang X., Zhou H., Liu J., Zhang S. (2016). Novel graphene/Au-CdS:Eu composite-based electrochemiluminescence immunosensor for cancer biomarker detection by coupling resonance energy transfer and enzyme catalytic reaction. J. Electroanal. Chem..

[17] Kim D., Kim J., Kwak C.H., Heo N.S., Oh S.Y., Lee H., Lee G.-W., Vilian A.T.E., Han Y.-K., Kim W.-S. (2017). Rapid and label-free bioanalytical method of alpha fetoprotein detection using LSPR chip. J. Cryst. Growth.

[18] Zhang F., Zhu J., Li J.-J., Zhao J.-W. (2015). A promising direct visualization of an Au@Ag nanorod-based colorimetric sensor for trace detection of alpha-fetoprotein. J. Mater. Chem. C.

[19] Chen H., Guan Y., Wang S., Ji Y., Gong M., Wang L. (2014). Turn-On Detection of a Cancer Marker Based on Near-Infrared Luminescence Energy Transfer from NaYF _4_: Yb, Tm/NaGdF _4_ Core–Shell Upconverting Nanoparticles to Gold Nanorods. Langmuir.

[20] Gao Y., Feng Y., Zhou L., Petti L., Wang Z., Zhou J., Xie S., Chen J., Qing Y. (2018). Ultrasensitive SERS-Based Immunoassay of Tumor Marker in Serum Using Au–Ag Alloy Nanoparticles and Ag/AgBr Hybrid Nanostructure. Nano.

[21] Zhai Y., Liu D., Jiang Y., Chen X., Shao L., Li J., Sheng K., Zhang X., Song H. (2019). Near-infrared-light-triggered photoelectrochemical biosensor for detection of alpha-fetoprotein based on upconversion nanophosphors. Sens. Actuators B Chem..

[22] Supchocksoonthorn P., Alvior Sinoy M.C., De Luna M.D.G., Paoprasert P. (2021). Facile fabrication of 17 β -estradiol electrochemical sensor using polyaniline/carbon dot-coated glassy carbon electrode with synergistically enhanced electrochemical stability. Talanta.

[23] Musa A., Kiely J., Luxton R., Honeychurch K. (2023). An Electrochemical Screen-Printed Sensor Based on Gold-Nanoparticle-Decorated Reduced Graphene Oxide–Carbon Nanotubes Composites for the Determination of 17-β Estradiol. Biosensors.

[24] Tong P., Kasuga Y., Khoo C.S. (2006). Liquid chromatographic-mass spectrometric method for detection of estrogen in commercial oils and in fruit seed oils. J. Food Compos. Anal..

[25] Zhong Q., Hu Y., Hu Y., Li G. (2012). Dynamic liquid–liquid–solid microextraction based on molecularly imprinted polymer filaments on-line coupling to high performance liquid chromatography for direct analysis of estrogens in complex samples. J. Chromatogr. A.

[26] Regan F., Moran A., Fogarty B., Dempsey E. (2003). Novel modes of capillary electrophoresis for the determination of endocrine disrupting chemicals. J. Chromatogr. A.

[27] Yilmaz B., Kadioglu Y. (2017). Determination of 17 β-estradiol in pharmaceutical preparation by UV spectrophotometry and high performance liquid chromatography methods. Arab. J. Chem..

[28] Alam F., Jalal A.H., Pala N. (2019). Selective Detection of Alcohol Through Ethyl-Glucuronide Immunosensor Based on 2D Zinc Oxide Nanostructures. IEEE Sens. J..

[29] Alam F., Jalal A.H., Sinha R., Umasankar Y., Bhansali S., Pala N. (2018). Sonochemically Synthesized ZnO Nanostructure-Based L-Lactate Enzymatic Sensors on Flexible Substrates. MRS Adv..

[30] Shinohara H., Tiwari A. (2015). Graphene: An Introduction to the Fundamentals and Industrial Applications.

[31] Prattis I., Hui E., Gubeljak P., Kaminski Schierle G.S., Lombardo A., Occhipinti L.G. (2021). Graphene for Biosensing Applications in Point-of-Care Testing. Trends Biotechnol..

[32] Abbas Q., Shinde P.A., Abdelkareem M.A., Alami A.H., Mirzaeian M., Yadav A., Olabi A.G. (2022). Graphene Synthesis Techniques and Environmental Applications. Materials.

[33] Whitener K.E., Sheehan P.E. (2014). Graphene synthesis. Diam. Relat. Mater..

[34] Lin J., Peng Z., Liu Y., Ruiz-Zepeda F., Ye R., Samuel E.L.G., Yacaman M.J., Yakobson B.I., Tour J.M. (2014). Laser-induced porous graphene films from commercial polymers. Nat. Commun..

[35] Liu X., Wang Y., Du Y., Zhang J., Wang Y., Xue Y., Zhao J., Ge L., Yang L., Li F. (2024). Laser-induced graphene (LIG)-based electrochemical microfluidic chip for simultaneous analysis of multiplex microRNAs. Chem. Eng. J..

[36] Feng Z., Geng Z., Pan S., Yin Y., Sun X., Liu X., Ge L., Li F. (2024). In situ patterning of nickel/sulfur-codoped laser-induced graphene electrode for selective electrocatalytic valorization of glycerol. Appl. Catal. B Environ. Energy.

[37] Liu X., Cheng H., Zhao Y., Wang Y., Li F. (2022). Portable electrochemical biosensor based on laser-induced graphene and MnO2 switch-bridged DNA signal amplification for sensitive detection of pesticide. Biosens. Bioelectron..

[38] Ghosh D., Tabassum R., Sarkar P.P., Rahman M.A., Jalal A.H., Islam N., Ashraf A. (2024). Graphene Nanocomposite Ink Coated Laser Transformed Flexible Electrodes for Selective Dopamine Detection and Immunosensing. ACS Appl. Bio Mater..

[39] Tsai J.-Z., Chen C.-J., Settu K., Lin Y.-F., Chen C.-L., Liu J.-T. (2016). Screen-printed carbon electrode-based electrochemical immunosensor for rapid detection of microalbuminuria. Biosens. Bioelectron..

[40] Ding L., Li Q., Zhou D., Cui H., An H., Zhai J. (2012). Modification of glassy carbon electrode with polyaniline/multi-walled carbon nanotubes composite: Application to electro-reduction of bromate. J. Electroanal. Chem..

[41] Zhu J., Song W., Peng J., Yin Y., Xu B., Wang C. (2022). Microwave thermally expanded graphene/polyaniline conductive paste for elaborate conductive pattern and conductive polyester fabric fabrication via screen printing. J. Coat. Technol. Res..

[42] Campos M., Miziara T.A., Cristovan F.H., Pereira E.C. (2014). Investigations of the electrical conduction mechanisms of polyaniline-DBSA/poly (acrylonitrile-butadiene styrene) blends. J. Appl. Polym. Sci..

[43] Navarchian A.H., Joulazadeh M., Karimi F. (2014). Investigation of corrosion protection performance of epoxy coatings modified by polyaniline/clay nanocomposites on steel surfaces. Prog. Org. Coat..

[44] Nayak R., Shetty P., Selvakumar M., Rao A., Rao K.M. (2022). Formulation of new screen printable PANI and PANI/Graphite based inks: Printing and characterization of flexible thermoelectric generators. Energy.

[45] Das J., Debnath A., Deb K., Saha B. (2023). Pressure Sensors Painted on Flexible Cellulose Substrates from Polyaniline-Based Conductive Ink. ACS Appl. Electron. Mater..

[46] Lee C.-W., Tsai H.-I., Lee W.-C., Huang S.-W., Lin C.-Y., Hsieh Y.-C., Kuo T., Chen C.-W., Yu M.-C. (2019). Normal alpha-fetoprotein hepatocellular carcinoma: Are they really normal?. J. Clin. Med..

[47] Rahman M.A., Pal R.K., Islam N., Freeman R., Berthiaume F., Mazzeo A., Ashraf A. (2023). A Facile Graphene Conductive Polymer Paper Based Biosensor for Dopamine, TNF-α, and IL-6 Detection. Sensors.

[48] Liu C., Liu T. (2023). A graphene-assisted electrochemical sensor for detection of alpha-fetoprotein in serum. Int. J. Electrochem. Sci..

[49] Giannetto M., Mori L., Mori G., Careri M., Mangia A. (2011). New amperometric immunosensor with response enhanced by PAMAM-dendrimers linked via self assembled monolayers for determination of alpha-fetoprotein in human serum. Sens. Actuators B Chem..

[50] Giannetto M., Elviri L., Careri M., Mangia A., Mori G. (2011). A voltammetric immunosensor based on nanobiocomposite materials for the determination of alpha-fetoprotein in serum. Biosens. Bioelectron..

[51] Xu Y.Y., Bian C., Chen S., Xia S. (2006). A microelectronic technology based amperometric immunosensor for α-fetoprotein using mixed self-assembled monolayers and gold nanoparticles. Anal. Chim. Acta.

[52] Jothi L., Jaganathan S.K., Nageswaran G. (2020). An electrodeposited Au nanoparticle/porous graphene nanoribbon composite for electrochemical detection of alpha-fetoprotein. Mater. Chem. Phys..

[53] Wang H., Li H., Zhang Y., Wei Q., Ma H., Wu D., Li Y., Zhang Y., Du B. (2014). Label-free immunosensor based on Pd nanoplates for amperometric immunoassay of alpha-fetoprotein. Biosens. Bioelectron..

[54] Lindon J.C., Tranter G.E., Koppenaal D. (2016). Encyclopedia of Spectroscopy and Spectrometry.

[55] Li J., Liu S., Yu J., Lian W., Cui M., Xu W., Huang J. (2013). Electrochemical immunosensor based on graphene–polyaniline composites and carboxylated graphene oxide for estradiol detection. Sens. Actuators B Chem..

[56] Wang Y., Luo J., Liu J., Li X., Kong Z., Jin H., Cai X. (2018). Electrochemical integrated paper-based immunosensor modified with multi-walled carbon nanotubes nanocomposites for point-of-care testing of 17β-estradiol. Biosens. Bioelectron..

[57] Zhang Y., Li J., Wang Z., Ma H., Wu D., Cheng Q., Wei Q. (2016). Label-free electrochemical immunosensor based on enhanced signal amplification between Au@ Pd and CoFe_2_O_4_/graphene nanohybrid. Sci. Rep..

[58] Lahcen A.A., Baleg A.A., Baker P., Iwuoha E., Amine A. (2017). Synthesis and electrochemical characterization of nanostructured magnetic molecularly imprinted polymers for 17-β-Estradiol determination. Sens. Actuators B Chem..

[59] Liu M., Ke H., Sun C., Wang G., Wang Y., Zhao G. (2019). A simple and highly selective electrochemical label-free aptasensor of 17β-estradiol based on signal amplification of bi-functional graphene. Talanta.

[60] Ming T., Wang Y., Luo J., Liu J., Sun S., Xing Y. (2019). Folding paper-based aptasensor platform coated with novel nanoassemblies for instant and highly sensitive detection of 17β-estradiol. ACS Sens..

[61] Jaradat H., Al-Hamry A., Nasraoui S., Barhoumi L., Ibbini M., Kanoun O. (2020). Immunosensor based on MWNT and Au Nanoparticles for detection of 17ß-estradiol in pg/mL. Proceedings of the 2020 17th International Multi-Conference on Systems, Signals & Devices (SSD).

